# Progression of hearing loss after LINAC-based stereotactic radiotherapy for vestibular schwannoma is associated with cochlear dose, not with pre-treatment hearing level

**DOI:** 10.1186/s13014-018-1202-z

**Published:** 2018-12-24

**Authors:** A. van Linge, R. van Os, N. Hoekstra, B. Heijmen, L. Stienstra, A. Dallenga, J. Wolbers, A. Mendez Romero

**Affiliations:** 1 Department of Otorhinolaryngology and Head & Neck surgery, Erasmus MC, Postbus 2040, 3000 Rotterdam, CA Netherlands; 2 Department of Radiotherapy, Academic Medical Center, Postbus 22660, 1100 Amsterdam, DD Netherlands; 3 Department of Radiotherapy, Erasmus MC Cancer Institute, Postbus 2040, Rotterdam, 3000 CA The Netherlands; 4 Department of Neurosurgery, Erasmus MC, Postbus 2040, Rotterdam, 3000 CA The Netherlands

**Keywords:** Vestibular schwannoma, Fractionated stereotactic radiotherapy, Radiosurgery, Hearing loss

## Abstract

**Background:**

Although stereotactic radiotherapy (SRT) for vestibular schwannoma has demonstrated excellent local control rates, hearing deterioration is often reported after treatment. We therefore wished to assess the change in hearing loss after SRT and to determine which patient, tumor and treatment-related factors influence deterioration.

**Methods:**

We retrospectively analyzed progression of hearing loss in patients with vestibular schwannoma who had received stereotactic radiosurgery (SRS) or fractionated stereotactic radiotherapy (FSRT) as a primary treatment between 2000 and 2014. SRS had been delivered as a single fraction of 12 Gy, and patients treated with FSRT had received 30 fractions of 1.8 Gy. To compare the effects of SRS and FSRT, we converted cochlear doses into EQD_2_. Primary outcomes were loss of functional hearing, Gardner Robertson (GR) classes I and II, and loss of baseline hearing class. These events were used in Kaplan Meier plots and Cox regression. We also calculated the rate of change in Pure Tone Average (PTA) in dB per month elapsed after radiation—a measure we use in linear regression—to assess the associations between the rate of change in PTA and age, pre-treatment hearing level, tumor size, dose scheme, cochlear dose, and time elapsed after treatment (time-to-first-audiogram).

**Results:**

The median follow-up was 36 months for 67 SRS patients and 63 months for 27 FSRT patients. Multivariate Cox regression and in linear regression both showed that the cochlear V90 was significantly associated with the progression of hearing loss. But although pre-treatment PTA correlated with rate of change in Cox regression, it did not correlate in linear regression. The time-to-first-audiogram was also significantly associated, indicating time dependency of the rate of change. None of the analysis showed a significant difference between dose schemes.

**Conclusions:**

We found no significant difference between SRS and FSRT. As the deterioration in hearing after radiotherapy for vestibular schwannoma was associated with the cochlea V90, restricting the V90 may reduce progression of hearing loss. The association between loss of functional hearing and baseline PTA seems to be biased by the use of a categorized variable for hearing loss.

## Background

An increasing number of studies have shown excellent local control after stereotactic radiotherapy (SRT) for vestibular schwannoma. [1–3]. There are two modalities for delivering SRT: single-fraction stereotactic radiosurgery (SRS) and fractionated stereotactic radiotherapy (FSRT). As both have been reported to lead to local control rates in the range of 93–100%, the focus has now shifted towards reducing toxicity.

While progression of hearing loss is often reported after SRT [4–9], there is no consensus on the factors that influence hearing outcome. Several papers [10–12], but not all [13], have reported that hearing outcome is significantly associated with age, tumor size and pre-treatment hearing level. Little is known about the influence of cochlear dose on hearing deterioration. Although most studies have not found a significant association between maximum cochlear dose and hearing deterioration [11, 14, 15], some have reported that hearing deterioration was significantly associated with mean cochlear dose [11, 14]; with the volume of the cochlea receiving at least 90% of the prescribed dose (V90) [8]; or with the volume of the cochlea receiving at least 5.3 Gy in a single dose [12].

Another area of uncertainty is whether FSRT yields better hearing preservation rates than SRS. A recent systematic review of clinical trials concluded that the evidence for better hearing preservation in vestibular schwannoma patients treated with FSRT is inconclusive [3]. This may be due to the lack of randomized trials comparing the two dose schemes. The only study that tried to address this issue in a randomized setting was closed prematurely and was modified into an observational study [16]. While it reported better hearing preservation after FSRT, it did not perform a cochlear DVH analysis. Neither did it have sufficient potential control for known baseline factors such as NF2 and tumor size. However, many other studies have not reported a significant difference [5, 6, 17, 18].

Many studies have analyzed hearing loss with a time-to-event analysis, using the preservation of functional hearing or preservation of the baseline hearing class as outcome variable. The hearing classifications used most commonly are the Gardner Robertson (GR) classification, and the classification of the American Academy of Otolaryngology – Head and Neck Surgery (AAO-HNS). Patients retaining GR class I or II, which correspond to AAO-HNS classes A or B, are considered to have preserved functional hearing. The reported rates of long-term functional hearing preservation after irradiation differ widely, from 31 to 94% [4].

Although the term functional hearing is an attractive concept, the limits of audiological functioning are not well defined by the limits of GR class I and II. Even when hearing loss exceeds 50 dB hearing may still be serviceable in the presence of good speech perception, but a drop in speech discrimination below 70% impedes audiological function at any level of pure tone average (PTA).

As better identification of factors that influence hearing loss would help physicians to optimize treatment planning and to achieve better hearing outcomes, we wished to identify factors that influence the rate of change in hearing after SRT. Hypothesizing that associations predicting the change in hearing after SRT might be identified through the use of continuous variables such as PTA rather than a dichotomous variable such as functional hearing classes, we used the change in PTA over time in our analysis, alongside the time-to-event analysis to analyze preservation of functional hearing class and preservation of GR baseline class.

## Methods and materials

### Study aim

The purpose of our study was to assess the change in hearing after SRS and FSRT in vestibular schwannoma patients, and to investigate patient, tumor, and treatment-related factors that influence hearing loss.

### Study design

This study was performed as a retrospective, single-institution study. The study protocol was approved by our local Medical Ethics Review Board.

### Patient enrollment

We reviewed all patients with unilateral vestibular schwannoma who had been treated with stereotactic radiotherapy at the Department of Radiation Oncology at Erasmus University Hospital between 1 January 2000 and 31 December 2014. The diagnosis of vestibular schwannoma had been based on the clinical presentation and the typical radiological appearance of a cerebellopontine angle (CPA) tumor upon gadolinium-enhanced magnetic resonance imaging (MRI). We excluded the following patients: those who had had previous interventions for vestibular schwannoma, those with neurofibromatosis type 2, and those who had not been audiometrically assessed less than 6 months before treatment. Unlike many other studies, we included all patients who had had measurable hearing before treatment; this enabled us to investigate the effect of treatment irrespective of pre-treatment hearing. We defined measurable hearing as PTA ≤ 100 dB and Speech Discrimination Score (SDS) > 0%, which corresponds to GR classes 1 through 4. Ninety-four patients of the 225 who had been treated were eligible for inclusion.

### Treatment allocation

All patients had been discussed in a multidisciplinary tumor board attended by a radiation oncologist, a neurosurgeon, an otolaryngologist and a specialized nurse practitioner. Treatment with SRT had been recommended for tumors touching or displacing the brain stem or cerebellar peduncle, and for tumors that progressed during wait and scan policy. The choice of technique had then been based on tumor size. Tumors extending up to 3 cm in the CPA cistern were preferably treated with SRS, whereas larger tumors were treated with FSRT. In the early years of the study period, FSRT was preferred to SRS for patients with functional hearing, independent of tumor size. Later on, this practice was abandoned on the basis of new publications [5, 6, 18], which did not find that FRST led to confirm a better hearing result of FSRT. In the event of symptoms due to trigeminal nerve or brain stem compression, a surgical intervention was recommended.

### Audiometry

Pure tone and speech audiometry had been performed in all patients less than 6 months before treatment and up to 10 years after its completion. We calculated PTA on the basis of the masked bone conduction responses at 500, 1000, 2000 and 4000 Hz. As the maximum phoneme score of “Consonant Vowel Consonant” words in percentages had been used for the SDS, we then classified according to the GR hearing classification [19]. GR hearing classes I and II represent functional hearing.

### Radiotherapy technique

During treatment, patients receiving SRS had been were immobilized using the invasive Brown-Roberts-Wells frame (Radionics, Integra NeuroSciences, Burlington, Massachusetts, USA), and with the relocatable Gill-Thomas-Cosman frame (Radionics) for FSRT. Until March 2014, treatments were delivered with a conventional LINAC (Varian 2300 CD, Varian Medical Systems, Palo Alto, California, USA) using 6 MV photons. From March 2014 onwards, SRS treatments were delivered with Cyberknife (Accuray Inc., Sunnyvale, California, USA) using a thermoplastic mask for immobilisation.

Using the immobilization system, a computerized tomography (CT) planning scan was acquired. These images were fused with MRI and this dataset was used for target delineation. The planning target volume (PTV) was defined as the area of contrast enhancement on T1-weighted MRI with no margin for SRS and with 2 mm margin for FSRT. The organs at risk considered were cochlea, brainstem, trigeminal nerve, optic nerves, optic chiasm, pituitary gland, and eyes. The cochlea was delineated on CT. The method of delineation was discussed with a neuroradiologist. All delineations were performed and reviewed by two of the authors (NH, AMR). For single-dose treatments, a dose of 12 Gy was prescribed at the 80% isodose surrounding the PTV. For fractionated treatments, a dose of 54 Gy was prescribed at the 100% isodose with the 95% isodose surrounding the PTV. FSRT was delivered in 30 daily fractions of 1.8 Gy over a period of 6 weeks.

### Follow-up

After treatment, patients were followed with MRI, audiometry and clinical evaluation. A post-treatment MRI and audiogram after SRT were planned at 12 months and thereafter at 2, 3, 5, 7 and 10 years. Not all patients complied with the full follow-up schedule.

### Outcome measures and variables

In the current study, our primary outcome was the progression of hearing loss after treatment. We compared three different ways to define hearing deterioration. First, deterioration was defined in the usual way, as loss of functional hearing (baseline GR classes I or II). Next, it was defined as loss of the baseline GR hearing class (class GR I to IV). In our third analysis, hearing loss was not categorized, but progression was measured as the rate of change in PTA in dB per month. This rate was calculated by dividing the difference between the PTA of the pre-treatment audiogram and each post-treatment audiogram by the number of months that had elapsed since SRT.

We evaluated the relationship between progression of hearing loss with patient, tumor, and treatment-related factors: age, pre-treatment hearing level, tumor volume, tumor diameter in the CPA, fractionation scheme, and cochlear doses.

To compare SRS and FSRT, we use the linear quadratic formula to convert cochlear doses into equivalent 2-Gy-fraction doses (EQD_2_) [11]:

1 $$ {\mathrm{EQD}}_2=D\frac{d+\left(\alpha /\beta \right)}{2+\left(\alpha /\beta \right)} $$

where EQD_2_ is the dose in 2-Gy-fractions that is biologically equivalent to the total dose D that was actually administered in fractions of d Gy [20]. The α/β ratio is a measure of cell death and cell repair, and can be assessed. For the cochlea, we used an α/β ratio of 2 [11]. We measured the maximum and mean cochlear dose. To calculate the volume of cochlea that had received at least 90% of the prescribed dose (V90) we converted 90% of the FSRT prescribed dose to an EQD_2._ Next, we sought to establish the equivalent dose delivered with SRS. We could then calculate the volume of the cochlea that had received this EQD_2_ in both the FSRT and SRS treatments.

### Data analysis

One-way ANOVA analysis was used to evaluate the differences between the FSRT and the SRS populations. The relationship between the change in PTA and the change in SDS was tested with the Wilcoxon Signed Rank test.

After categorizing hearing according to the Gardener Robertson grading, we used the Kaplan Meier method to assess the preservation of useful hearing and the preservation of baseline hearing class. Factors that could be predictive of an increase in GR class were evaluated with the Cox proportional hazard model for univariate and multivariate analysis.

The change in PTA is a continuous outcome measure that allows univariate and multivariate linear regression analysis of the monthly rate of PTA increase. We used the step-backward method to optimize both multivariate models, this method removes factors from the model that are not significant, until only significant factors remain. The factors removed are rated non-significant, without reporting a hazard ratio (HR), regression coefficient (RC) or *p*-value. Relationships with *p* < 0.05 were considered to be significant.

We performed all analyses using the statistical programs R (version 2.13.0) and IBM SPSS (version 23).

## Results

### Patient characteristics

Between 01 January 2000 and 31 December 2014, 225 patients had been treated for vestibular schwannoma with either technique. Ninety-four patients fulfilled the criteria for inclusion in this study. Patients were excluded due to the absence of an audiometric assessment taken less than 6 months before treatment or due to a pre-treatment speech-discrimination score equal to zero.

Sixty-seven patients had been treated with SRS and 27 with FSRT. Our treatment algorithm caused differences between the two groups (Table 1). Patients who had been treated with SRS were older, had been in a higher pre-treatment GR class, and had had smaller tumors. Median follow-up had been 36 months for the SRS group and 63 months for the FSRT group. None of the patients had required an additional intervention for vestibular schwannoma.

**Table 1:** Patient, tumor and treatment characteristics

	SRS group (67 patients)			FSRT group (27 patients)			ANOVA *p*-value
	*N* (%)	mean/ median	range	*N* (%)	mean/ median	range	
Patients							
Age	67	58/59	34–80	27	48/51	27–79	< 0.001
Gender							0.2
Male	32			9			
Female	35			18			
Hearing							
GR baseline class							0.01
1	7 (10,4)			13 (48.1)			
2	30 (44.8)			7 (25.9)			
3	28 (41.8)			5 (18.5)			
4	2 (2.9)			2 (7.4)			
Δ PTA (dB/month)		1.35/0.95	−0.86-11.13		2.9/1.65	−0.65-28	0.05
PTA pre-treatment (dB)		48.2/46.3	1.3–96		36/34	3.7–94	0.02
SDS pre-treatment (%)		78/85	18–100		83/94	3–100	0.38
Tumors							
Tumor side							
Left	37			14			0.8
Right	30			13			
Tumor volume Pre-treatment (ml)		3.0/2.36	0.37–12.82		7.4/6.8	0.24–20.1	0.003
Tumor diameter CPA (cm)		1.7/1.56	0.64/3.34		2.4/2.56	0.67/4.22	< 0.001
Tumor diameter IAC (cm)		0.88/0.93	0.15/1.39		0.83/0.93	0/1.41	0.453
Radiation							
Cochlear volume (ml)		0.17/0.17	0.08–0.31		0.22/0.22	0.17–0.36	< 0.001
Max cochlear dose EQD2		3578/3990	448–6693		4623/4840	3428–5258	< 0.001
Mean cochlear dose EQD2		1054/736	114–3211		3569/3781	1355–4866	< 0.001
V90 EQD2 (mm3)		4/0	0–51		65/50	0–256	< 0.001
Follow-up time (months)		37/36	4.8–121		61/63	8.3–123	< 0.001

All radiation doses are given in Gy in EQD_2_

### Pre-treatment hearing

**Fig. 1:**
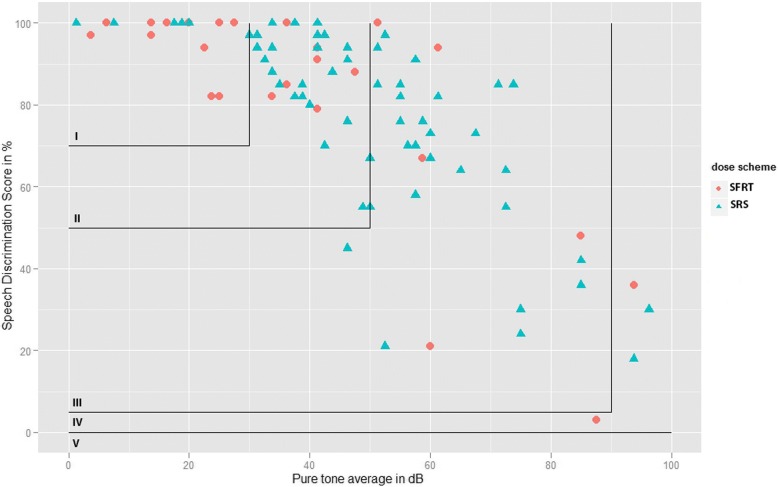
Speech discrimination score plotted against the pure tone average for all patients before treatment. The correlation coefficient is − 0.752. Overlay of the Gardner Robertson classification grid

Figure 1 shows the scattergram of pre-treatment hearing levels. The PTA and SDS are significantly correlated (*r* = − 0.752, *p* < 0.0001).

Categorization of hearing according to the Gardner Robertson classification showed that the patients’ distributions over the hearing classes differed between dose schemes. Table 1 shows that 48.1% of FSRT patients had started out in hearing class I and that 25.9% had started in hearing class II. By contrast, while only 10.4% of those in the SRS group had started out in hearing class I, 44.8% had started in hearing class II.

### Loss of functional hearing

**Fig. 2:**
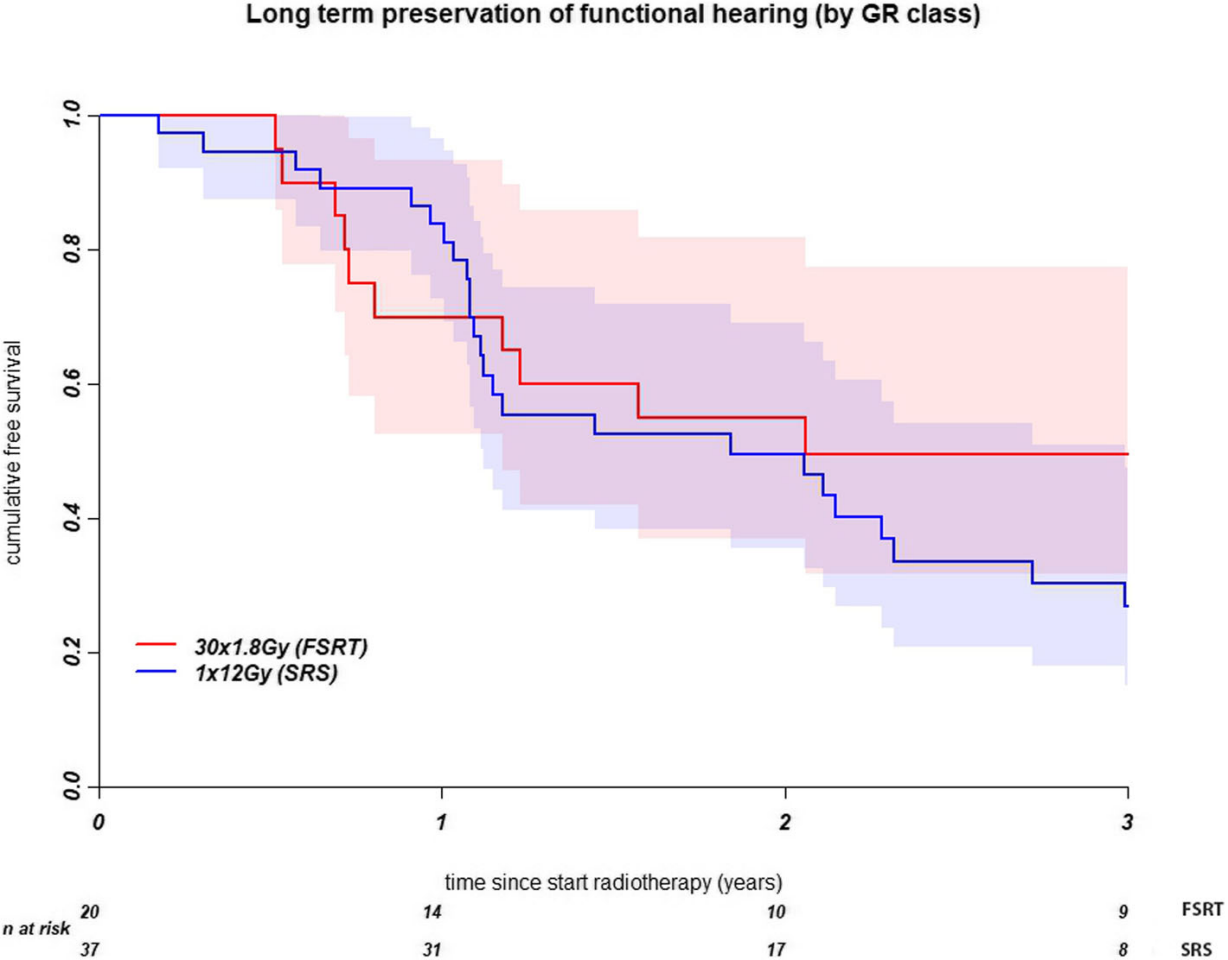
Kaplan-Meier curve for preservation of functional hearing (Gardner Robertson classes I and II) for stereotactic radiosurgery (SRS) and fractionated stereotactic radiotherapy (FSRT)

Before radiotherapy, 57 out of 94 patient had had hearing class I or II. Preservation of GR hearing class I or II had not differed significantly between the two treatment groups (Fig. 2, Table 2). One year after treatment, 84% of the SRS patients and 71% of the FSRT patients had preservation of useful hearing. At 3 years, useful hearing had been retained in 27% of the SRS patients and 50% of the FSRT patients.

**Table 2:** Univariate and multivariate Cox regression of loss of functional hearing in 57 patients

GR class I&II, *n* = 57	Univariate				Multivariate			
	Hr	95% CI Lower	95% CI Upper	*p*-value	HR	95% CI Lower	95% CI Upper	*p*-value
Age	1.03	1.005	1.06	0.017				n.s.
PTA pre treatment	1.072	1.04	1.11	< 0.001	1.081	1.045	1.118	< 0.001
Tumor diameter CPA (cm)	0.72	0.47	1.12	0.15				n.s.
Tumor diameter IAC (cm)	2.70	0.93	7.85	0.07				n.s.
Tumor Volume pre- treatment (ml)	0.94	0.86	1.03	0.17				n.s.
Dose scheme (0 = fract, 1 = rs)	2.05	1.01	4.17	0.05				n.s.
Max cochlear dose EQD_2_^a^	1	1	1	0.99				n.s.
Mean cochlear dose EQD_2_^a^	1	1	1	0.58				n.s.
V90 cochlea EQD2 ^b^	1.01	1.00	1.013	0.027	1.011	1.004	1.017	0.001
Cochlear volume (ml)	1.0	0.99	1.003	0.26				n.s.
time-to-first-audiogram (after treatment)	1.0	0.95	1.04	0.85				n.s.

*n.s.* Not significant, 95% CI = 95% confidence interval, *PTA* Pure tone average

^a^per cGy,

^b^per mm^3^

Univariate Cox regression showed that loss of functional hearing was significantly associated with the V90, pre-treatment PTA, and age. After multivariate regression, pre-treatment PTA and V90 remained significant factors.

### Loss of baseline GR class

**Fig. 3:**
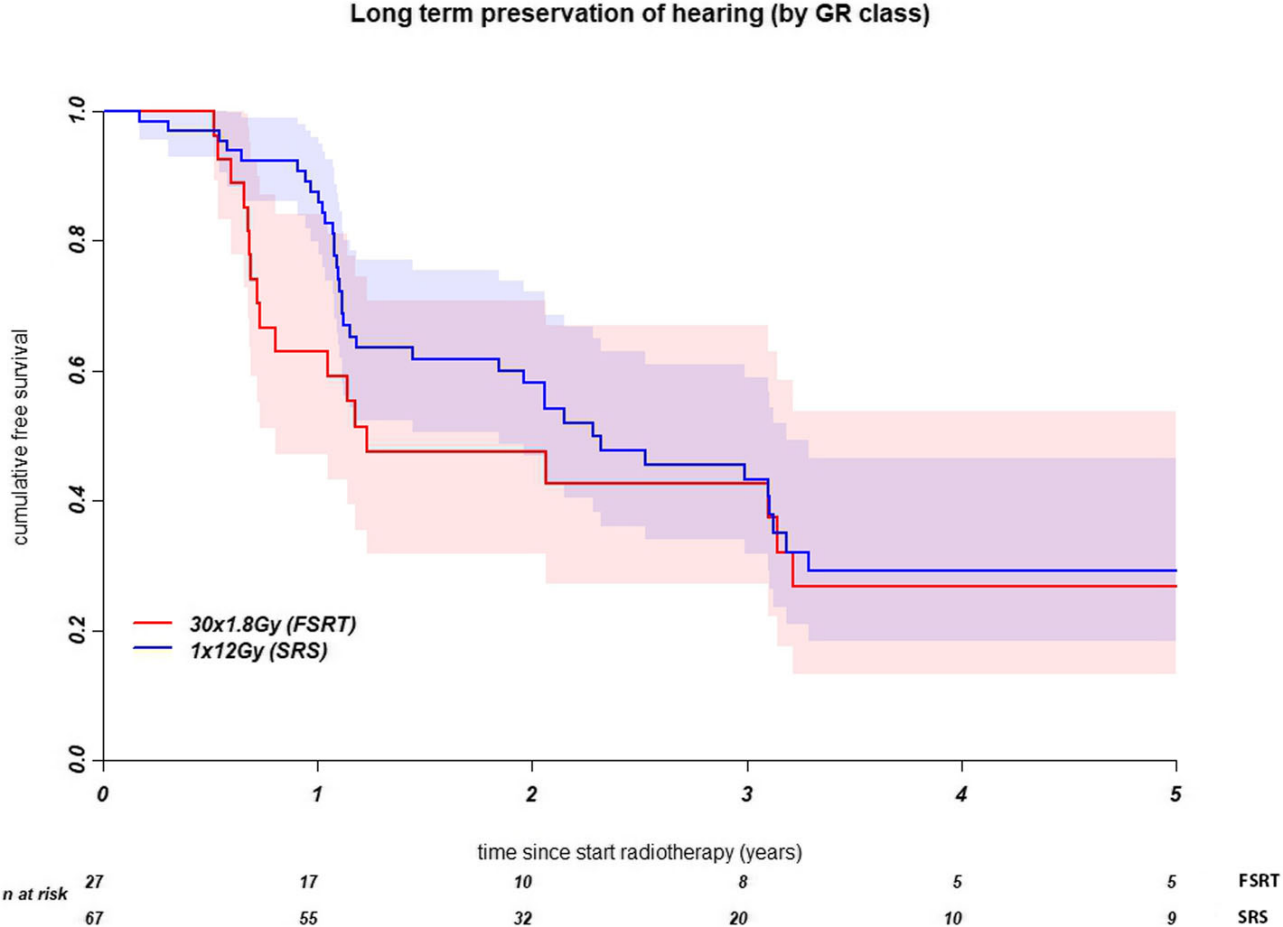
Kaplan-Meier curve for the preservation of Gardner Robertson class for stereotactic radiosurgery (SRS) and fractionated stereotactic radiotherapy (FSRT)

Preservation of baseline GR class did not differ significantly between the two treatment groups (Fig. 3, and Table 3). At 1 year after treatment, baseline GR class had been preserved in 88% of the SRS patients and 63% of the FSRT patients. At 3 years, it had been retained in respectively 43 and 39%.

**Table 3:** Univariate and multivariate Cox regression analysis of increase in baseline GR class in 94 patients (Multivariate backward method)

Total study group	Univariate analysis				Multivariate analysis			
*n* = 94	HR	95% CI Lower	95% CI Upper	*p*-value	HR	Lower 95% CI	Upper 95% CI	*p*-value
Age	0.98	0.97	1.000	0.079				n.s
PTA pre- treatment	0.99	0.98	1.002	0.13	0.99	0.97	1.0	0.023
Tumor diameter CPA (cm)	1.06	0.75	1.52	0.74				n.s.
Tumor diameter IAC (cm)	1.13	0.44	2.92	0.80				n.s.
Tumor volume pre- treatment (ml)	1.02	0.95	1.09	0.63				n.s.
Dose scheme (0 = FSRT, 1 = SRS)	0.84	0.48	1.48	0.54	2.19	0.95	5.038	0.067
Max cochlear dose cGy EQD_2_^a^	1	1	1	0.33				n.s
Mean cochlear dose cGy EQD_2_^a^	1	1	1	0.27				n.s.
V90 cochlea EQD_2_^b^	1.008	1.002	1.014	0.006	1.013	1.006	1.021	0.001
Cochlear volume (ml)	0.998	0.993	1.003	0.37				n.s.
Time-to-first-audiogram (after treatment)	0.98	0.96	1.001	0.07				

*n.s*. Not significant, 95% *CI* 95% confidence interval, *PTA* Pure tone average

^a^per cGy,

^b^per mm^3^

Univariate Cox regression showed a significant association of loss between baseline GR hearing class and the V90. Pre-treatment PTA was significantly associated with loss of baseline hearing class only after multivariate regression. Age did not show an association.

### PTA increase per month

**Fig. 4:**
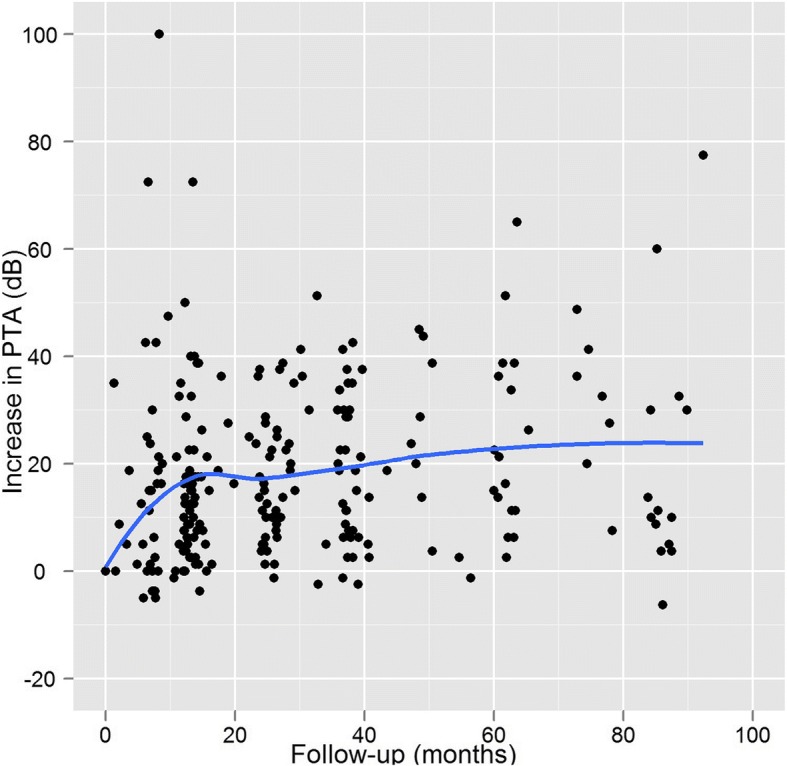
Differences for all patients between pre- and post-treatment PTA for each follow-up audiogram

**Fig. 5:**
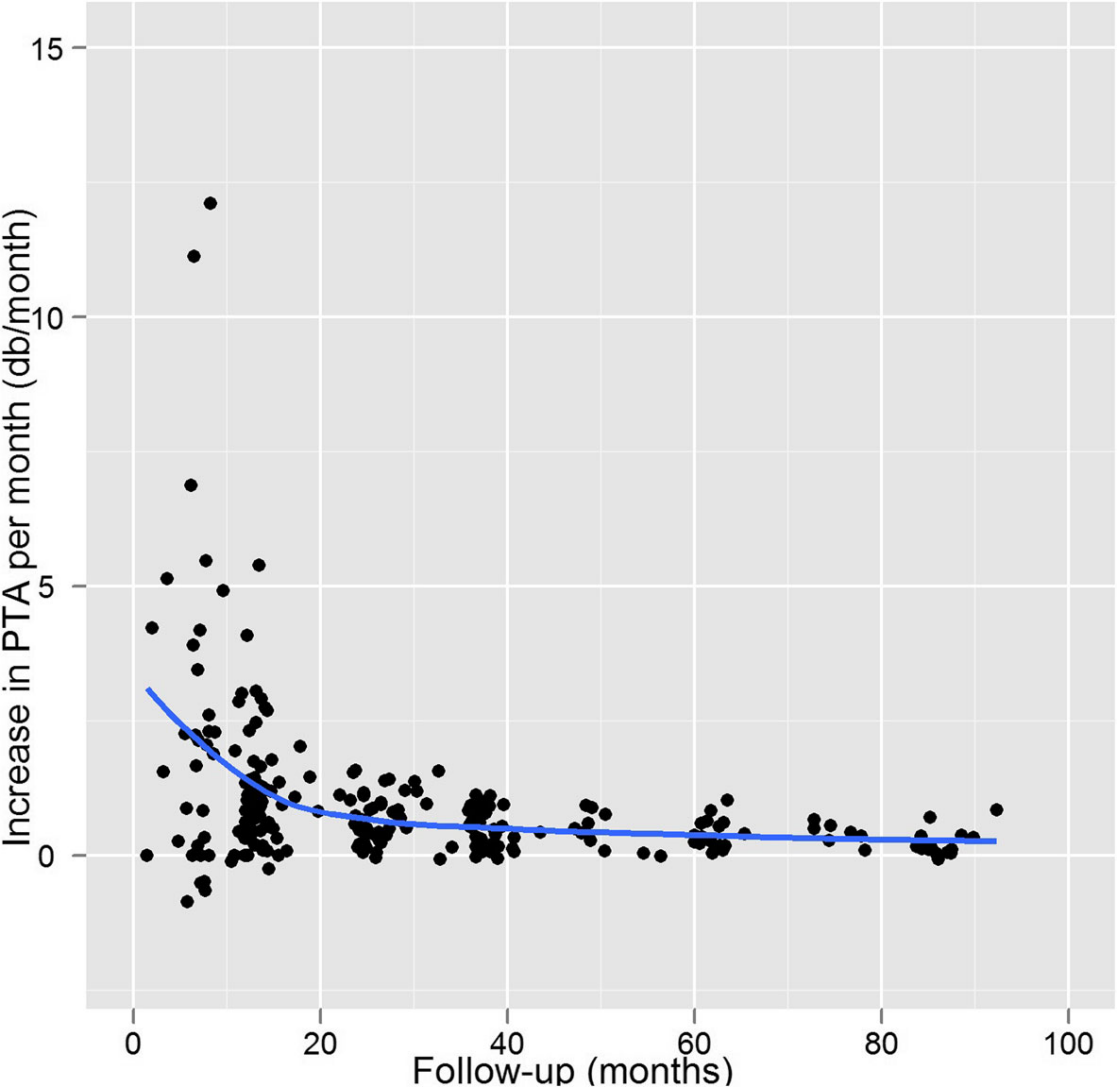
Slope of PTA increase in dB/month for each follow-up audiogram; all patients

Figure 4 shows the differences per patient and per follow-up audiogram between pre-treatment PTA and post-treatment PTA. This shows that hearing loss continued to increase up to 90 months, so hearing did not stabilize within this period. Figure 5 shows the monthly rate of hearing deterioration after treatment. While PTA had initially increased steeply in patients with an early audiogram, audiograms after longer intervals showed a smaller change per time unit, proportional to the time elapsed.

To compare results between patients despite the different follow-up times, we included the time elapsed after treatment as a variable in the analysis. This was useful, as the follow-up schedule had not been adhered to for all patients. As the greatest change occurred shortly after treatment, we only included patients whose first audiogram had been taken within 2 years of follow up. As the date of audiometry clustered around the scheduled follow-up dates, we used 21 months as our cut-off for inclusion in this analysis, i.e., between the 18-month and 24-month follow up. Seventy-seven patients had had an audiometric assessment within the first 21 months (Table 4); up to the first audiogram, the mean PTA increase had been 1.74 dB per month. Both the univariate and multivariate analysis showed the PTA increase per month to be significantly associated with age, time-to-first-audiogram and V90(insert Table 4).

**Table 4:** Univariate and multivariate linear regression of deterioration of the PTA in 77 patients with audiograms within 21 months after treatment

Cases with first audiometric follow up < = 21mnths *n* = 77	Univariate				Multivariate			
	RC	95% CI Lower	95%CI Upper	Univar *p*-value	RC	95% CI Lower	95% CI Upper	Multivar *p*-value
Age	0.072	0.005	0.139	0.035	0.078	0.030	0.261	0.012
PTA pre- treatment	0	−0.041	0.041	0.99				n.s.
Tumor diameter CPA (cm)	0.649	−0.482	1.779	0.26				n.s.
Tumor diameter IAC (cm)	2.66	−0.064	5.38	0.06				n.s.
Tumor volume pre- treatment (ml)	0.21	0.003	0.416	0.046				n.s.
Dose scheme (0 = FSRT, 1 = SRS)	−1.31	−3.09	0.461	0.145				n.s.
Max cochlear dose cGy EQD_2_^a^	0.000	0.000	0.001	0.264				n.s.
Mean cochlear dose cGy EQD_2_^a^	0.001	0.000	0.001	0.005				n.s.
V90 cochlea EQD_2_^cb^	0.025	0.009	0.041	0.003	0.024	0.009	0.039	0.002
Cochlear volume (ml)	−0.003	− 0.020	0.014	0.74				n.s.
Time-to-first-audiogram (after treatment)	−0.365	− 0.585	− 0.145	0.001	− 0.302	− 0.508	−0.096	0.005

*n.s*. Not significant, 95% *CI* 95% confidence interval, *PTA* Pure tone average

Outcome variable: ΔPTA in dB/month. Multivariate backward method. 0 = reference

^a^per cGy,

^cb^per mm3

## Discussion

After stereotactic irradiation for vestibular schwannoma, hearing had deteriorated markedly. FSRT and SRS did not differ significantly with regard to the preservation of functional hearing and baseline GR class. The cochlear V90 was significantly associated with hearing deterioration. Within GR classes I & II, patients with a better PTA remained in their hearing class for longer than patients whose hearing had already diminished. This was not duplicated in the linear regression analysis.

### Method of analyzing hearing loss

Although preservation of functional hearing is a common criterion in the analysis of hearing change in vestibular schwannoma treatment, it has inherent limitations.

First, whereas the concept of “preservation” suggests a stable final outcome, we found—as Fig. 4 shows—that hearing had continued to decrease. After 2 years of follow-up, the findings of Yomo et al. were similar: an ongoing annual loss of 1.8 dB [21]. Second, “functional hearing” is not adequately defined by GR classes I and II, as speech discrimination of over 80% is needed to understand spoken language. Limiting the analysis to patients in GR classes I and II therefore has two limitations: it includes a number of patients in the analysis whose hearing is no longer good enough to understand spoken language, and it excludes patients in GR classes III whose speech perception is sufficient for them to benefit from a hearing aid (Fig. 1). Although this cutoff point between serviceable and non-serviceable hearing is an arbitrary one, any other cutoff point would also be arbitrary. Rather than limiting this analysis to patients with GR classes I and II hearing, we extended it to all patients with measurable speech perception.

At any given moment after radiation, the hearing level depends on pre-treatment hearing and on the rate of decline up to that time. Only the rate of decline is influenced by treatment. As hearing continues to decrease, any absolute PTA outcome in dB needs to be coupled to the corresponding length of follow up; and a rate also couples the amount of dB lost to a time interval. Therefore we used a rate as outcome measure, rather than an absolute outcome in dB. We performed longitudinal analysis of the increase in PTA in dB per month. The rate of hearing decline is not a novel outcome measure: Yomo et al. used longitudinal analysis to compare the change in PTA between pre- and post-treatment hearing loss [21]. For our analysis of the rate of change of the PTA, we assumed that the change in PTA would be linear. This may be valid only for short periods after treatment, as we found that hearing deterioration accelerated shortly after radiation, and then slowed. (Figs. 4 and 5). For this reason, we included only audiograms acquired within 2 years of follow up. This ensured that we included only the period with the greatest changes, and also that we used a comparable interval for all patients. Patients without an audiogram within this timeframe were excluded from the analysis.

The multivariate analysis showed time to first audiogram to be significantly associated with PTA increase per month (RC -0.37). This sustains our hypothesis that the rate of hearing deterioration decreases over time after treatment—a finding that is in agreement with others, who also described a rapid decline during the initial period after treatment (ranging from 6 to 10 months [6] to 2 years [7]), with a more gradual decline in hearing after that. After 2 years, the hearing level did not quite stabilize (see Fig. 4). In their series of patients, Yomo et al. also found a slow rate of continued decline after 2 years [21]. Further research with standardized timing of follow-up would make it possible to analyze in greater detail how hearing progresses after radiotherapy. The progression we describe here suggests that all patients should have audiometry immediately before therapy, followed by audiometry every 6 months for the first 2 years after therapy, and then at increasing intervals.

### Hearing loss and pre-treatment hearing level

The Cox analysis of functional hearing showed an association between pre-treatment hearing class and the loss of functional hearing. This suggests that better hearing is less sensitive to deterioration—a common finding in patients with pre-treatment GR class I and II [7, 9, 11, 14–16]. If, rather than a change in hearing class, the change in PTA per month, is used as the outcome measure, there is no association between pre-treatment hearing and deterioration of hearing level. Patients with a similar rate of change in PTA, but with a better baseline PTA, retain their baseline GR class I & II longer, and in these cases the GR classification is relatively insensitive to changes in hearing.

### Difference between FSRT and SRS

Our finding of no significant difference between SRS and FSRT is in agreement with the findings of several other studies that directly compared the two treatment modalities. These studies used various outcome measures to evaluate hearing: preservation of GR class, preservation of GR I or II, and patient-reported hearing function [5, 6, 17, 18]. Only Andrews et al. [16] found a significant difference, with a rate of hearing preservation that was 2.5 higher in the patients treated with FSRT, However, Andrews used Gammaknife radiosurgery and prescribed the treatment dose to the 50% isodose, while many other studies used LINAC SRS and prescribed the dose to the 80 to 100% isodose. This may have influenced the dose distribution to the cochlea and acoustic nerve, and, as a result, may have had an impact on hearing outcome [22]. However, he did not perform a DVH analysis. Similar percentages of hearing preservation were found by Coughlin et al. in their large review comparing hearing preservation in Gammaknife radiosurgery and linear accelerator technologies [23].

### Hearing loss and cochlear dose

In the literature there has been considerable debate on the influence of cochlear dose on hearing loss, and also on the method of measuring it. Although volumetric assessment of cochlear dose seems more relevant than a point dose [14], Thomas et al. found a significance between patients whose hearing had been preserved and those in whom it had deteriorated, both in volumetric values V90, V80, V50, and in point doses Dmax and Dmin.

Our finding that there was no significant association between the preservation of functional hearing and the maximum cochlear dose is in agreement with several other studies [11, 14, 15]. While we did find a significant association in the univariate analysis of the mean cochlear dose and the PTA increase per month, the cochlear V90 carried a much stronger statistical association.

After investigating the cochlear V90, Rasmussen et al. and Thomas et al. [8, 24] both reported a significant relationship of the cochlear V90 with loss of hearing preservation after FSRT. We investigated whether the V90 was associated with hearing loss in both FSRT and SRS. For the SRS patients, the V90 was often zero. This was the consequence of the steep dose gradient generated with this technique and the absence of PTV margins around the tumor. In agreement with Rasmussen, the V90 was the strongest predictor of hearing loss. It retained significance in the multivariate analysis both in the Cox analysis and in the linear regression.

### Study limitations

As not all patients had complied with the follow-up schedule, our study has several limitations. Patients had had their post-treatment assessments at various points in the schedule, a problem we dealt with by using the time to first audiogram as a variable that represented the time dependence of hearing deterioration. The sooner after radiation the audiogram had been taken, the faster the rate of deterioration had been. This suggests that normal tissue-complication modeling is needed for these stereotactic treatments.

The groups of patients who had received FSRT and SRS differed in several respects, such as tumor size, PTV margin and dose distribution. The two latter factors may influence the V90, specifically when the intracanalicular tumor component is located close to the cochlea. As our use of the linear-quadratic approach (EQD_2_ formulae) was intended to make the different fractionation schedules comparable between the groups, readers interpreting our results should remember that this is just a model.

The two groups of patients receiving FSRT and SRS differed with regard to several factors, such as tumor size, PTV margin and the conformality of the dose distributions. The two latter factors may influence the value obtained for the cochlea V90, especially in tumors with an intracanalicular component that extends until the inner ear. By using the linear quadratic model approach (EQD2 formulae), we aimed to make the two schemes we had applied comparable. When interpreting these results, it should be realized that this approach has some limitations. For large fraction doses such as 12 Gy, the calculations may be less certain than for low doses per fraction. Another point that deserves special attention is the α/β selected to perform the EQD2 calculations. While the value of 2 was chosen on the basis of the published literature, it could also be argued that 3 could be also have been used, and that these different values may have had some small impact on the results [25]. Last but not least, we should point out that the calculation of the V90 was carried out taking 90% of the prescribed dose delivered with FSRT as a reference. This approach was chosen because the experience published on V90 and hearing loss has been reported on FSRT. The consequence is that although we were able to compare results on V90 with other published series, we need to remember that the EQD2 value corresponding to the 90% of the prescribed dose delivered with FSRT will not represent exactly the 90% of the prescribed dose delivered with SRS.

## Conclusions

Our results confirm that hearing deteriorates after SRT for vestibular schwannoma. In our patient population, the most rapid decline had occurred shortly after treatment.

We found that the effects of FSRT and SRS on hearing change had not differed significantly.

The cochlear V90 was associated with progression of hearing loss. Restricting the V90 during radiotherapy treatment planning may help to reduce progression of hearing loss.

Better baseline hearing does not protect against changes in PTA. Although a protective effect may be apparent when hearing was evaluated dichotomously, it was not observed when the PTA was evaluated as a continuous variable.

## Abbreviations

AAO-HNS: The American Academy of Otolaryngology – Head and Neck Surgery
CPA: Cerebellopontine angle
CT: Computerized tomography
DVH: Dose volume histogram
EQD_2_: Equivalent 2-Gy-fraction doses
FSRT: Fractionated stereotactic radiotherapy
GR: Gardner Robertson
HR: Hazard ratio
IAC: Internal acoustic canal
MRI: Magnetic resonance imaging
NTCP: Normal tissue complication probability
PTA: Pure tone average (average hearing level of 0.5, 1, 2, 4 kHz)
PTV: Planning target volume
RC: Regression coefficient
SDS: Speech discrimination score
SRS: Stereotactic radiosurgery
SRT: Stereotactic radiotherapy
Vx: Volume of the organ of interest receiving at least x % of the prescribed dose
